# Whole genome sequencing of the multidrug-resistant *Chryseobacterium indologenes* isolated from a patient in Brazil

**DOI:** 10.3389/fmed.2022.931379

**Published:** 2022-07-28

**Authors:** Marcelo Silva Folhas Damas, Roumayne Lopes Ferreira, Emeline Boni Campanini, Gabriela Guerrera Soares, Leslie Camelo Campos, Pedro Mendes Laprega, Andrea Soares da Costa, Caio César de Melo Freire, André Pitondo-Silva, Louise Teixeira Cerdeira, Anderson Ferreira da Cunha, Maria-Cristina da Silva Pranchevicius

**Affiliations:** ^1^Departamento de Genética e Evolução, Universidade Federal de São Carlos, São Carlos, SP, Brazil; ^2^Laboratório Central de Saúde Pública do Tocantins, Palmas, TO, Brazil; ^3^Programa de Pós-graduação em Odontologia e Tecnologia Ambiental, Universidade de Ribeirão Preto, Ribeirão Preto, SP, Brazil; ^4^Department of Vector Biology, Liverpool School of Tropical Medicine, Liverpool, United Kingdom; ^5^Centro de Ciências Biológicas e da Saúde, Biodiversidade Tropical - BIOTROP, Universidade Federal de São Carlos, São Carlos, Brazil

**Keywords:** *Chryseobacterium indologenes*, whole-genome sequencing, virulence and resistance genes, mobile genetic elements, neonatal intensive care unit, metabolic features

## Abstract

Chryseobacterium indologenes is a non-glucose-fermenting Gram-negative bacillus. This emerging multidrug resistant opportunistic nosocomial pathogen can cause severe infections in neonates and immunocompromised patients. This study aimed to present the first detailed draft genome sequence of a multidrug-resistant *C. indologenes* strain isolated from the cerebrospinal fluid of an infant hospitalized at the Neonatal Intensive Care Unit of Brazilian Tertiary Hospital. We first analyzed the susceptibility of *C. indologenes* strain to different antibiotics using the VITEK 2 system. The strain demonstrated an outstanding resistance to all the antibiotic classes tested, including β-lactams, aminoglycosides, glycylcycline, and polymyxin. Next, *C. indologenes* was whole-genome-sequenced, annotated using Prokka and Rapid Annotation using Subsystems Technology (RAST), and screened for orthologous groups (EggNOG), gene ontology (GO), resistance genes, virulence genes, and mobile genetic elements using different software tools. The draft genome contained one circular chromosome of 4,836,765 bp with 37.32% GC content. The genomic features of the chromosome present numerous genes related to cellular processes that are essential to bacteria. The MDR *C. indologenes* revealed the presence of genes that corresponded to the resistance phenotypes, including genes to β-lactamases (*bla*_*IND–*13_, *bla*_*CIA–*3_, *bla*_*TEM–*116_, *bla*_*OXA–*209_, *bla*_*VEB–*15_), quinolone (*mcb*G), tigecycline (*tet*(X6)), and genes encoding efflux pumps which confer resistance to aminoglycosides (*Ran*A/*Ran*B), and colistin (*Hly*D/*Tol*C). Amino acid substitutions related to quinolone resistance were observed in GyrA (S83Y) and GyrB (L425I and K473R). A mutation that may play a role in the development of colistin resistance was detected in lpxA (G68D). *Chryseobacterium indologenes* isolate harbored 19 virulence factors, most of which were involved in infection pathways. We identified 13 Genomic Islands (GIs) and some elements associated with one integrative and conjugative element (ICEs). Other elements linked to mobile genetic elements (MGEs), such as insertion sequence (ISEIsp1), transposon (Tn5393), and integron (In31), were also present in the *C. indologenes* genome. Although plasmids were not detected, a ColRNAI replicon type and the most resistance genes detected in singletons were identified in unaligned scaffolds. We provided a wide range of information toward the understanding of the genomic diversity of *C. indologenes*, which can contribute to controlling the evolution and dissemination of this pathogen in healthcare settings.

## Introduction

*Chryseobacterium indolog*enes is a non-motile, catalase-positive, oxidase-positive, indole-positive, non-glucose-fermenting Gram-negative bacilli (1) widely distributed in nature, but it is not normally present in the human microflora (2, 3). In humans, *C. indologenes* was isolated for the first time from a tracheal aspirate of a patient with ventilator-associated pneumonia (4). Although considered a relatively uncommon human pathogen, the number of hospital-acquired infections caused by *C. indologenes* has been increasing (5–8). Since *C. indologenes* can survive in inanimate objects, it may be isolated from hospital environments and cultured from specimens of sinks, indwelling vascular catheters, vials, feeding tubes, and other equipment that are in contact with fluids and water (9). Therefore, *C. indologenes* is considered a potential reservoir for various types of serious infections (8, 10), including pneumonia, meningitis, wound infection, intraabdominal infection, primary bacteremia, intravascular catheter–related bacteremia, and cellulitis (7, 11–14). Generally, major risk factors for *C. indologene* infection are patients with predisposing diseases, immunocompromised status, long-term broad-spectrum antibiotics treatment, and long-term hospitalization with indwelling devices (1, 15, 16).

Although the clinical significance and pathogenicity of *C. indologenes* are not well established (17, 18), *C. indologenes* is known to be naturally resistant to a wide variety of antibiotics including not only aminoglycosides, tetracyclines, chloramphenicol, macrolides, aminopenicillins, clindamycin, teicoplanin but also first-generation cephalosporins, aztreonam, ticarcillin-clavulanate, and carbapenems (10, 19–21). The most potent drugs reported against *C. indologenes* are quinolones (gatifloxacin and levofloxacin), minocycline, and trimethoprim-sulfamethoxazole (18, 22, 23); however, many studies show an alarming trend in resistance to those antibiotics (3, 9, 24).

Whole-genome sequencing (WGS) has become a well-established technique for high-resolution characterization of the genetic repertoire of bacterial pathogens, including antibiotic resistance, molecular epidemiology, and virulence (25). It is a promising technique for surveillance and monitoring infection control and outbreak cases of many microbial pathogens of interest in public health (26). Hence, we conducted the complete genome sequencing of MDR *C. indolo*genes to understand the genomic diversity and genes responsible for antibiotic resistance and virulence. To the best of our knowledge, there is no published data on *C. indologenes* whole-genome sequencing from the cerebrospinal fluid sample of a hospitalized infant in Tocantins, Brazil.

## Materials and methods

### Patient and bacterial isolate

*Chryseobacterium indologenes* was isolated from the cerebrospinal fluid (CSF) of an infant hospitalized at the Neonatal Intensive Care Unit (NICU) of Hospital Geral de Palmas, Palmas, Tocantins, Brazil. This isolate was initially identified as *C. indologenes* by a clinical microbiology laboratory using conventional methods. It was sent to the Central Laboratory of Public Health of Tocantins (LACEN/TO) for species confirmation and drug susceptibility testing. LACEN is a healthcare facility in the Brazilian Ministry of Health that receives samples for the surveillance of antimicrobial resistance.

### Bacteria information and antimicrobial susceptibility

Once the sample was received at LACEN, bacterial identification and drug susceptibility assays were performed by the Vitek 2 system (bioMérieux, Marcy-l’Etoile, France) and interpreted according to Clinical and Laboratory Standards Institute guidelines (27). *Chryseobacterium indologenes* isolate was tested for susceptibility against 16 antibiotics: ampicillin (AMP), ampicillin/sulbactam (SAM), piperacillin/tazobactam (TZP), cefuroximeaxetil (CXM-AX), cefoxitin (FOX), ceftazidime (CAZ), ceftriaxone (CRO), cefepime (FEP), ertapenem (ETP), imipenem (IPM), meropenem (MEM), amikacin (AMK), gentamicin (GEN), ciprofloxacin (CIP), tigecycline (TGC), and colistin (CST).

Minimum inhibitory concentration (MICs) of colistin and tigecycline was determined by the broth microdilution (BMD) method according to the EUCAST (28) recommendations. The results obtained with the VITEK 2 system were compared to those obtained by the BMD method.

Phenotypic detection for the production of carbapenemases was carried out by modified Hodge test, synergy test, and the ethylenediaminetetraacetic acid (EDTA) test under the CLSI guidelines (27) as described elsewhere (29–32). Multidrug-resistant (MDR) *C. indologenes* isolate was defined by non-susceptibility to at least one agent in three or more antibiotic categories (33).

### DNA isolation and genome sequencing

Genomic DNA extraction was done in an overnight culture using the Wizard^®^ Genomic DNA Purification Kit (Promega, Madison, WI, United States), according to the manufacturer’s instructions. The DNA extract concentration and purity were determined by measuring absorbance at wavelengths of 260 and 280 nm (NanoVue Plus; GE Healthcare Life Sciences, Marlborough, MA, United States). The integrity of genomic DNA was tested by way of electrophoresis. Bacterial DNA concentration was also measured fluorometrically (Qubit^®^ 3.0, kit Qubit^®^ dsDNA Broad Range Assay Kit, Life Technologies, Carlsbad, CA, United States). The sample from the isolate was prepared for sequencing using 1 ng of input genomic DNA. Nextera XT DNA Library Prep Kit (Illumina, San Diego, CA, United States) was used for library preparation. The libraries were amplified using a limited cycle PCR program. The PCR step adds Index 1(i7) adapters, Index 2 (i5) adapters, and sequences required for sequencing cluster generation. The purification of the amplified library was performed using 0.6x Agencourt AMPure XP beads (Beckman Coulter). For checking the library quality and size of fragmented DNA, the samples were evaluated on 1.5% electrophoresis agarose gel. The libraries were quantified with a fluorometric method Qubit^®^ 3.0 using kit Qubit^®^ dsDNA Broad Range Assay Kit, Life Technologies, Carlsbad, CA, United States) and normalized to 4 nM using a Standard Dilution Method. The libraries were pooled, denatured with 0.2 N NaOH, and diluted to the final concentration of 1.8 pM. A PhiX control was added to a final concentration of 1.5 pM. The run-length was a paired-end run of 75 cycles for each read (2 × 75), plus up to eight cycles each for two index reads.

### Genome assembly and annotation

Raw reads were assessed for quality using FastQC v.0.11.9, a quality control tool for high throughput sequence data,^1^ and filtered for quality, length, and adapter regions using TrimGalore! v.0.6.5, a wrapper tool specific for FastQC.^2^ The *de novo* genome assembly was made with SPAdes v.3.15.3 (“careful” and “cov-cutoff auto” options selected) (34) and SSPACE software (35). Plasmid detection and assembly attempts were made using PlasmidFinder 2.1^3^ (36) and PlasmidSPAdes (37), respectively. Scaffolds of less than 200 bp were discarded. We assessed the general statistics of the assembled genome using QUAST v5.0.2 (38). The graphical map of the circular genome was generated using CGView Server (39).

Genome annotations were conducted with Prokka v.1.14.5 annotation pipeline (40) and Rapid Annotation using Subsytems Technology (RAST) server v.2.0^4^ (41). Orthologous groups were analyzed using eggNOG mapper v2^5^ (42). Blast2GO (43) and Kyoto Encyclopedia of Genes and Genomes (KEGG)^6^ (44) were used for the determination of Gene Ontology (GO) annotation and gene role in metabolism, respectively.

### Phylogenetic inferences using 16S rRNA gene, average nucleotide identity, and DNA–DNA hybridization

We identified the *C. indologenes* 16S rRNA gene sequence from our genome annotation. We considered reference sequences of the 16S rRNA gene from other 14 *Chryseobacterium* species available at the GenBank database, including another *C. indologenes* strain. *Elizabethkingia miricola* was used as the outgroup. The accession numbers of these sequences are shown in the phylogeny. Nucleotide sequences were aligned using MAFFT v.7 (45). With the software MEGA-X (46), we estimated the best-fitting nucleotide model of substitution. This information was used to construct the phylogenetic tree by the Maximum Likelihood (ML) method. The robustness of branches was assessed by bootstrap analysis of 1,000 replicates (47).

The average nucleotide identity (ANI) values among our *C. indologenes* genome and other twelve *Chryseobacterium* genomes (Supplementary Table 1) were calculated using OrthoANI^7^ (48). From these same genomes, we calculated the *in silico* DNA--DNA hybridization (DDH)-analogous values using the GGDC v.3.0 tool^8^ (49). Heat maps of ANI and DDH were generated using CIMminer.^9^

### Comparative pan-genome analysis of *Chryseobacterium indologenes* strains

We constructed a whole-genome sequence-based phylogenetic tree considering our *C. indologenes* genome and other 15 *Chryseobacterium indologenes* genomes (Supplementary Table 2) using the online pipeline REALPHY v.1.13 (50) with default settings. The four closest strains of our *C. indologenes* were then analyzed with OrthoVenn2^10^ (51), a webserver used for genome-wide annotation and comparison of orthologous gene clusters. Bacterial Pangenome Analysis Pipeline (BPGA) (52) was used for the identification of the core, accessory, and unique genes, and their functional distribution in KEGG categories.

### Identification of antimicrobial resistance and virulence-associated genes

For the identification of antibiotic resistance genes, we used the AMRFinderPlus tool from NCBI (53), ResFinder 4.1 tool^11^ from DTU (54), and the Comprehensive Antibiotic Resistance Database (CARD; (55);^12^ RGI tool, with a 70% identity cutoff. We also performed BLASTp analysis against the ARG-ANNOT V6 database (56) with a 1E-5 *e*-value, > 50% identity, and > 90% query coverage cut-off. The results obtained from the KEGG functional annotation were also considered.

Putative virulence genes were predicted through BLASTp analysis against the Virulence Factor Database (VFDB) (57) using a 1E-5 *e*-value, > 50% identity, and > 90% coverage cut-off.

### Identifications of mutations associated with quinolone and colistin resistance

Genes responsible for colistin and quinolone resistance were identified through the rapid prokaryotic genome annotation (PROKKA). The amino acid sequences obtained from lpxA, lpxC, lpxD, and pmrC were manually analyzed for known mutations conferring resistance to colistin. *Acinetobacter* multispecies (WP_196075311.1) and *Acinetobacter baumanii* (SUU42982.1) lpxA sequences were manually aligned with our *C. indologenes* sequence (58). Other genes associated with polymyxin resistance, such as *pmr*B, *pmr*A, *pho*P, and *pho*Q were not found in the annotation executed by PROKKA.

The presence of a known mutation responsible for quinolone resistance on the *gyr*A, *gyr*B, and *par*C genes was investigated using BLASTp software of NCBI.^13^ The deduced amino acid sequences of the *C. indologenes* gyrA were aligned with *Chryseobacterium* multispecies (WP_027372477.1) and *Chryseobacterium indologenes* (QPQ51520.1). Amino acid sequence alignment for the gyrB. of *C. indologenes* was made with *Chryseobacterium aurantiacum* (WP_106916365.1), *Chryseobacterium joostei* (WP_076354057.1), *Chryseobacterium arachidis* (WP_072953039.1), and *Chryseobacterium taiwanense* (WP_039365989.1). Substitutions of amino acids in parC sequence described in the literature were not found in our *C. indologenes*.

### Genomic islands, insertion sequences, and other mobile genetic elements detection

IslandViewer 4 webserver^14^ (59) was used for the identification of the genomic island using FDAARGOS_379 as a reference strain. Insertion sequences, transposons, and integrons were predicted using ISFinder^15^ (60), TnCentral^16^ (61), and Integron Finder^17^ (62) webservers, respectively. CRISPR sequences were searched with CRISPRCas Finder^18^ (63) on default parameters. The PHASTER webserver^19^ (64) was used for the detection of phage-associated sequences in the genome. Integrative and conjugative elements (ICEs) and Integrative mobile elements (IMEs) were predicted with the ICEfinder tool^20^ (65) from the ICEberg 2.0 database using the default settings.

### Sequences accession number

The raw reads were submitted to Sequence Reads Archives,^21^ and the Bioproject accession id is PRJNA830910. Moreover, all the sequence data that we analyzed are related to this Bioproject id.

## Results

### Characteristics of the patient and antibiotic resistance profile of strain

Our *C. indologenes* strain presented resistance to all tested antibiotics, including β-lactams (Ampicillin, Ampicillin-Sulbactam, Piperacillin-Tazobactam, Cefuroximeaxetil, Cefoxitin, Ceftazidime, Ceftriaxone, Cefepime, Ertapenem, Imipenem, Meropenem); aminoglycosides (Amikacin, Gentamicin); quinolones (Ciprofloxacin); glycylcycline (Tigecycline); and polymyxin (Colistin) (Table 1). The isolate was defined as multidrug-resistant (MDR). However, the following antibiotics were not included in the MDR classification: amikacin and gentamicin (aminoglycosides); ampicillin and ampicillin-sulbactam (aminopenicillins); imipenem, meropenem, and ertapenen (carbapenems). This was because *C. indologenes* is intrinsically resistant to these antibiotics (18, 19, 66, 67).

**TABLE 1:** Phenotyping and antibiotic resistance genes found within the *Chryseobacterium indologenes* genome.

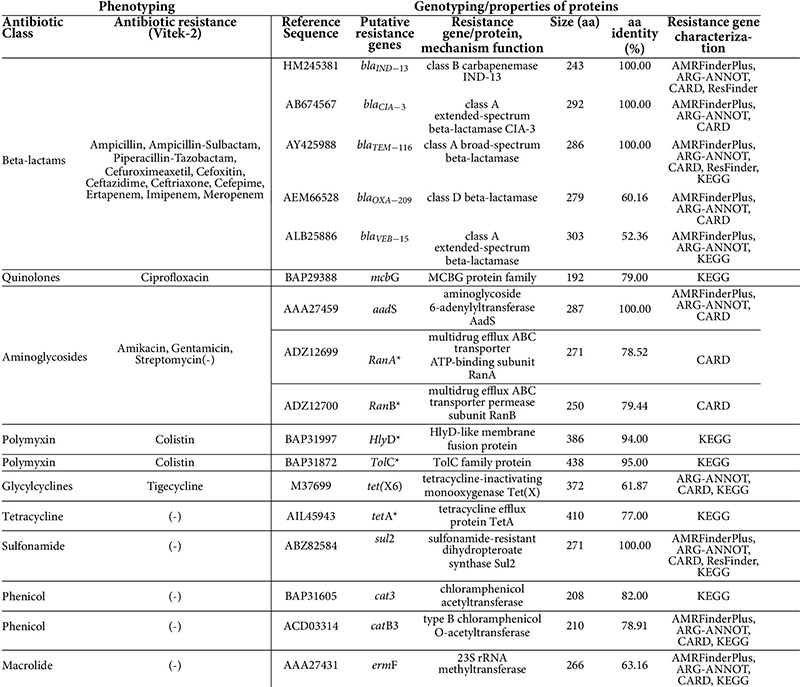

*Multidrug efflux pumps. (-) Susceptibility testing was not performed.

### General features of the *Chryseobacterium indologenes* genome

**FIGURE 1:**
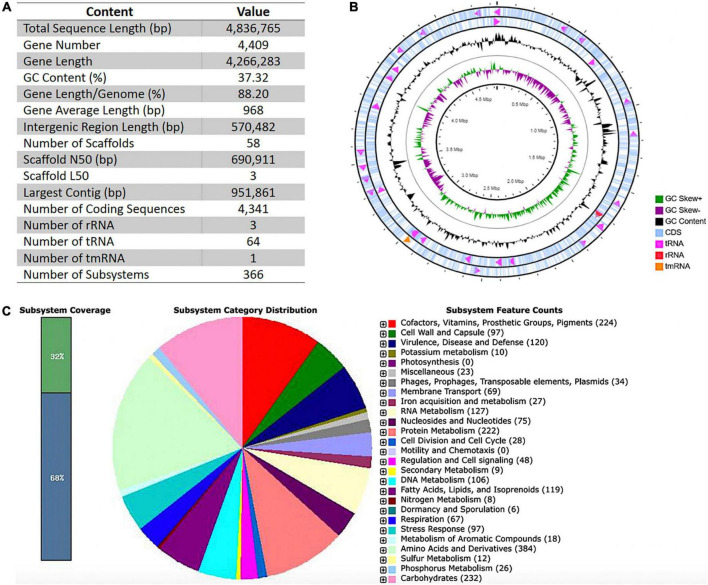
Basic data of Whole-Genome Sequencing, circular representations, and subsystem category distributions of *Chryseobacterium indologenes*. (A) Assembly and annotation statistics. (B) Circles are numbered from 1 (outer) to 4 (inner). The outer two circles represent the coding sequence (CDS), transfer ribonucleic acid (tRNA), ribosomal ribonucleic acid (rRNA), and transfer-messenger RNA (tmRNA). The third circle shows the GC content (black). The fourth circle demonstrates the GC skew curve (positive GC skew, green; negative GC skew, violet). (C) The genome of *C. indologenes* annotated by the Rapid Annotation System Technology (RAST) server was classified into subsystems and categories. The green part in the bar chart at the leftmost position corresponds to the percentage of proteins included. The pie chart and count of the subsystem features in the right panel show the percentage distribution and category of the subsystems.

The total length of the draft genome assembled was 4,836,765 bp, comprising one circular chromosome, with 37.32% G + C content. We detected a COIRNAI plasmid replicon but could not assemble any plasmid. The statistics of assembly and annotation are shown in Figure 1A. The assembly comprised 58 scaffolds and 4,409 genes that covered 88.20% of the genome. Of these genes, 4,341 were predicted to be coding sequences (CDSs). Of the 68 RNA genes predicted, three were rRNAs, 64 transfer RNAs (tRNAs), and one transfer-messenger RNA (tmRNA) (Figures 1A,B). The RAST analysis showed that the genome of *C. indologenes* comprises 366 subsystems that could be classified into 27 categories. The five most significant categories in this genome were “amino acids and derivatives” that accounted for 384 genes, followed by “carbohydrates” (232 genes), “cofactors, vitamins, prosthetic groups, pigments” (224 genes), “protein metabolism” (222 genes), and “virulence, disease, and defense” (120). In the category of “virulence, disease, and defense,” 94 genes were found to be related to “resistance to antibiotics and toxic compounds,” including beta-lactamase (13 genes), multidrug resistance efflux pumps (13 genes), multidrug resistance, tripartite systems found in gram-negative bacteria (12 genes), resistance to fluoroquinolones (5 genes), and aminoglycoside adenylyltransferases (1 gene) (Figure 1C).

### Orthologous genes and gene ontology of *Chryseobacterium indologenes*

**FIGURE 2:**
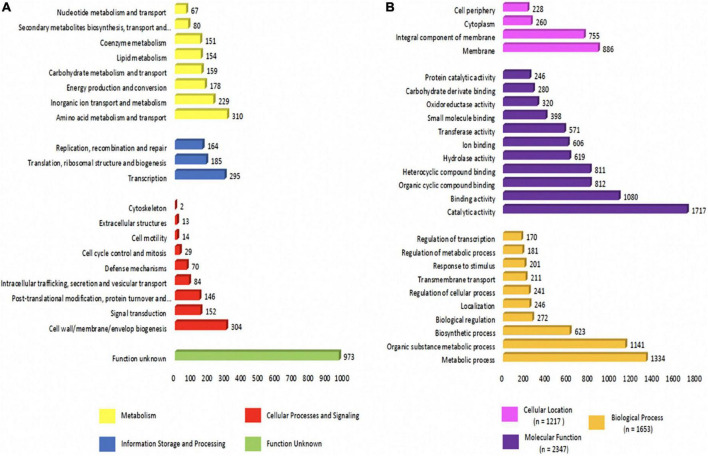
Functional annotations of *Chryseobacterium indologenes* strain. (A) Clusters of orthologous groups (COGs) based on functional annotation using eggNOG-mapper. (B) Distribution of gene ontology (GO).

The distribution of protein-coding genes into the Cluster of Orthologous Groups (COG) functional category using EggNog resulted in a total of 3,759 genes. The majority of known protein-coding genes were related to “amino acid metabolism and transport” (*n* = 310; 8.25%), followed by those associated with “cell wall/membrane/envelope biogenesis” (*n* = 304; 8.09%), and “transcription” (*n* = 295; 7.85%). The number of genes associated with defense mechanisms was 70 (1.86%), and with “unknown functions” it was 973 (25.88%) (Figure 2A).

The Gene Ontology (GO) distribution of our *C. indologenes* strain showed a total of 2,900 genes, which accounted for 65.77% of the entire encoded genes. Of these, 7,460 GO terms were associated with “molecular function,” 4,620 GO terms with “biological process,” and 2,129 GO terms with “cellular location.” The GO distribution showed that within the molecular function, the main subcategories were “catalytic activity (1.717 GO terms, 23.02%), “binding activity” (1.080 GO terms, 14.48%), and “organic cyclic compound binding” (812 GO terms, 10.88%). Among the biological process, most of the genes were characterized to the subcategories like “metabolic process” (1.334 GO terms, 28.87%), “organic substance metabolic process” (1.141 GO terms, 24.70%), and “biosynthetic process” (623 GO terms, 13.48%); Within the cellular location, the most highly assigned GO terms were “membrane” (*n* = 886, 41.62%), “integral component of membrane” (*n* = 755, 35.46%), and “cytoplasm” (*n* = 260, 12.21%) (Figure 2B).

### Phylogenetic position and similarity of whole genomes

**FIGURE 3:**
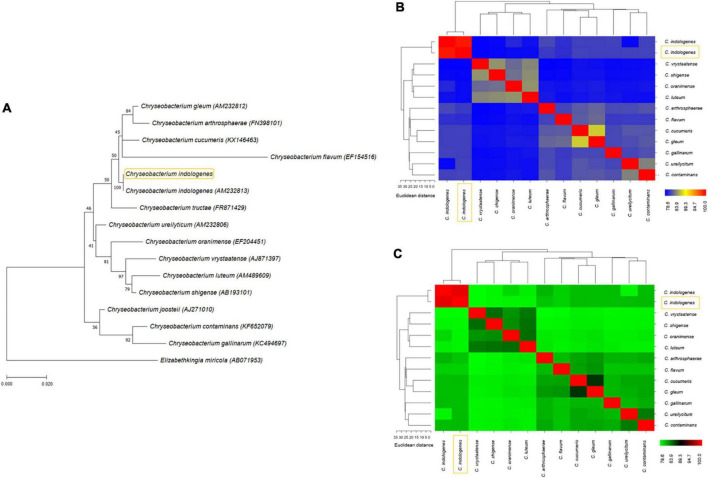
(A) Phylogenetic tree based on 16S rRNA showing the relationship among our *C. indologenes* strain and other reference sequences of 16S rRNA obtained from public databases. The number next to the node is the statistical bootstrap value. In brackets are GenBank accession numbers of the 16S rRNA genes. The scale bar indicates 0.020 substitutions per nucleotide position. The heat maps of average nucleotide identity (ANI) (B) and *in silico* DNA–DNA hybridization (DDH) (C) between our *C. indologenes* genome and twelve *Chryseobacterium* genomes. The yellow box represents our strain.

A phylogenetic tree based on the 16S rRNA sequence was constructed with fourteen 16S rRNA reference sequences related to *Chryseobacterium* and one 16S rRNA reference sequence of *Elizabethkingia miricola* as an outgroup. This aimed to define the evolutionary position of our *C. indologenes* strain. Analyses revealed that our *C. indologenes* was most closely related to *Chryseobacterium indologenes* (AM232813). Nevertheless, it also showed a similarity between different species, including *Chryseobacterium cucumeris* (KX146463), *Chryseobacterium arthrosphaerae* (FN398101), *Chryseobacterium gleum* (AM232812), and *Chryseobacterium flavum* (EF154516) (Figure 3A).

The genomic similarity between our sample and the twelve *Chryseobacterium* species that had complete genome sequences in the NCBI (Figure 3A) was evaluated using *in silico* ANI (Supplementary Table 3) and DDH (Supplementary Table 4) analyses (Figures 3B,C) to confirm the species relatedness inferred from the phylogenetic tree and ensure an accurate assignment at the species level. The ANI analysis found that our *C. indologenes* and *C. indologenes* (GCF_900460995.1) had a high similarity of 99.06% (Figure 3B). The DDH value between *C. indologenes* and *C. indologenes* (GCF_900460995.1) was 91.7% (Figure 3C). Therefore, both ANI and DDH analysis indicated that our *C. indologenes* strain belongs to the *C. indologenes* species.

### PhyloGenetic tree and genetic relatedness

**FIGURE 4:**
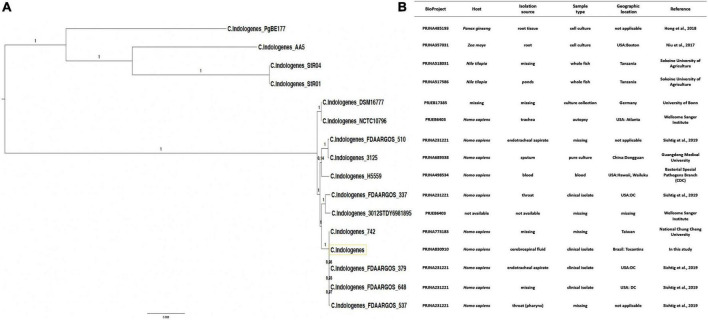
(A) Phylogenetic tree showing the evolutionary similarity between *C. indologenes* and other 15 selected strains of *Chryseobacterium indologenes*. (B) Genome Assembly and Annotation report (https://www.ncbi.nlm.nih.gov/genome/browse/#!/prokaryotes/14653/) of 16 strains of *C. indologenes*. The yellow box represents our strain.

We performed a phylogenetic analysis based on *C. indologenes* genomes downloaded from the NCBI database. The resulting tree topology was assessed to identify genetic relatedness between our *C. indologenes* isolate and 15 *C. indologenes* strains (Figure 4A). Our analysis showed that our *C. indologenes* strains is more closely related to *Chryseobacterium indologenes* 742 (SAMN22445227), *Chryseobacterium indologenes* FDAARGOS_379 (SAMN07312423), *Chryseobacterium indologenes* FDAARGOS_537 (SAMN10163232), and *Chryseobacterium indologenes* FDAARGOS_648 (SAMN11056363) (Figure 4A). The characteristics of the 16 *C. indologenes* strains are shown in Figure 4B.

### Orthologous groups and Kyoto Encyclopedia of Genes and Genomes distribution in the closest *Chryseobacterium indologenes* genomes

**FIGURE 5:**
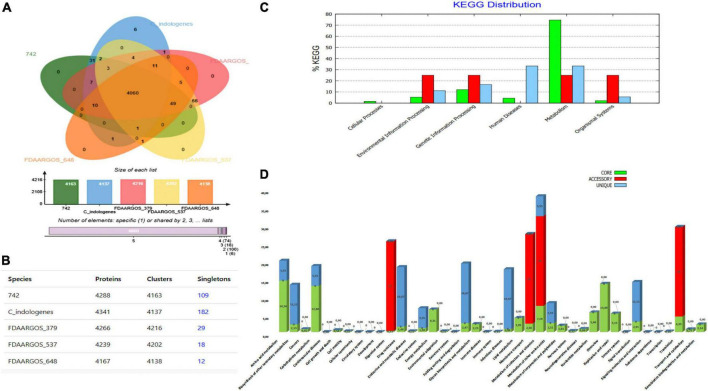
Comparative genomic analysis. (A) Venn diagram and bar chart showing the numbers of unique and shared orthologous genes in the five most closely related *C. indologenes*. (B) Number of proteins, clusters, and singletons. (C) KEGG pathway classification in core, accessory, and unique genomes. (D) Distribution of KEGG pathway classification.

Using OrthoVenn web server, comparison and annotation of orthologous gene clusters were performed between our *C. indologenes* and its four most closely related neighbors. Data indicates that there are 4,060 core-conserved genes shared by all five strains and a total of 6 strain-specific gene clusters in our *C. indologenes* strain. These unique genes were related to metal ion transport, response to cadmium ion, and copper ion transport (Figure 5A). The meanings of these unique gene clusters are not clear, and further studies are needed to better understand the functions of these singular genes. Our *Chryseobacterium indologenes* strain contained the highest number of singletons (*n* = 182), followed by *C. indologenes* 742 (*n* = 109), *C. indologenes* FDAARGOS_379 (*n* = 29), *C. indologenes* FDAARGOS_537 (*n* = 18), and *C. indologenes* FDAARGOS_648 (*n* = 11) (Figure 5B). We also found *sul*2, *bla*_*TEM–*116_, *aad*S, *bla*_*VEB–*15_, *bla*_*OXA–*209_, *cat*B e *erm*F resistance genes in singletons of our *C. indologenes* strain.

The KEGG functional distribution showed that genes were associated mainly with “metabolism” and were the most abundant in core (74.62%) compared to unique (33.33%) and accessory (25%) genomes (Figure 5C). In the core genome, genes were mainly related to “amino acid metabolism” (14.36%), “overview” (13.49%), and “carbohydrate metabolism” (12.88%) (Figure 5D). In the accessory genome, “environmental information processing,” “genetic information processing,” “metabolism,” and “organismal systems” accounted for similar portions (25%) (Figure 5C). These genes were associated with “digestive system” (25%), “membrane transport” (25%), “metabolism of cofactors and vitamins” (25%), and “translation” (25%) (Figure 5D). In the unique genome, “metabolism” (33.33%), “human diseases” (33.33%), and “genetic information processing” (16.67%) accounted for most genes (Figure 5C). These genes were mainly associated with “folding, sorting, and degradation” (16.67%), “infectious diseases” (16.67%), and “drug-resistance” (16.67%) (Figure 5D). We found 27 genes related to “drug resistances” in the unique genome. Of these, 11 were related to beta-lactam resistance, 11 were associated with cationic antimicrobial peptide resistance, and 5 were vancomycin resistance genes. Our data suggest that antibiotic resistance plays an important function in all of the *C. indologenes* strains analyzed.

### Resistome of *Chryseobacterium indologenes*

**FIGURE 6:**
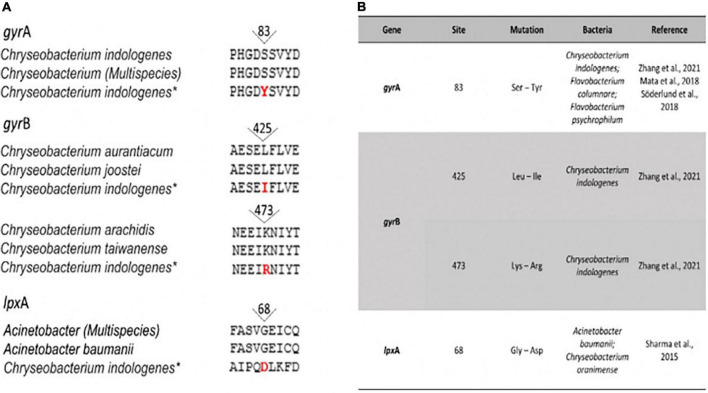
(A) Amino acid sequence alignment for the GyrA QRDR (Ser83Tyr) of *Chryseobacterium ureilyticum*, *Chryseobacterium* (Multispecies), Chryseobacterium indologenes*; GyrB QRDR (Leu425Ile) of *Chryseobacterium aurantiacum*, *Chryseobacterium joostei*, Chryseobacterium indologenes* and GyrB QRDR (Lys473Arg) of *Chryseobacterium arachidis*, *Chryseobacterium taiwanense*, Chryseobacterium indologenes*; and mutations in lpxA (Gly68Asp) of *Chryseobacterium* (Multispecies), Acinetobacter baumanii, Chryseobacterium indologenes*. (B) Known mutations in genes conferring resistance to quinolones (GyrA and GyrB QDDR) and colistin (lpxA). *Strain isolated in this study.

The whole-genome sequence analysis of *C. indologenes* corroborated with the phenotypic analyses, which revealed several antibiotic resistance-related genes (Table 1 and Figure 6). These antibiotic resistance genes included 5 β-lactamases (*bla*_*IND–*13_, *bla*_*CIA–*3_, *bla*_*TEM–*116_, *bla*_*OXA–*209_, *bla*_*VEB–*15_), 1 quinolone gene (*mcb*G), 1 tigecycline gene [tetracycline-inactivating monooxygenase *tet*(X6)], 1 RanA and 1 RanB genes that encode an efflux pump which confers resistance to aminoglycosides, 1 outer-membrane protein (*Tol*C) and 1 gene of membrane fusion protein (*Hly*D) genes that mediate resistance to colistin antibiotic (Table 1). Additionally, our *in silico* analysis found genes that can confer resistance to tetracycline [*tet*(A)] (68), streptomycin (*aad*S) (69), phenicol (*cat*3, *cat*B3) (70), sulfonamide (*sul*2) (71), and macrolides (*erm*F) (72); however, our strains were not tested phenotypically to these antibiotics (Table 1).

Gene functions annotated using the RAST/PROKKA tool identified *gyr*A, *gyr*B, and *lpx*A gene, in which amino acid substitution can also confer antibiotic resistance for fluoroquinolone and colistin, respectively. Amino acid substitutions related to quinolone resistance were also found at positions 83 of DNA gyrase subunit A (gyrA: Ser83Tyr) and at positions 425 and 473 of DNA gyrase subunit B (gyrB: Leu425Ile and Lys473Arg). Mutations that may play a role in the development of colistin resistance were found at lpxA (G68D) of *C. indologenes* (Figures 6A,B).

### Virulence-associated genes detection

*Chryseobacterium indologene* strain revealed the presence of 19 virulence factorsthat were associated with adhesion (elongation factor *Tu*, *ilp*A), stress response (*kat*A, *kat*G, *clp*P, *and gro*L), environmental adaptation (*ure*G, *ure*B), biofilm formation (*clp*P, *gal*E), metabolism (*clp*E), motility (*rff*G), polysaccharide biosynthesis (tviB), O-antigen nucleotide sugar biosynthesis (*wlb*B), O-antigen-synthesis (*rfb*A), capsular polysaccharide synthesis and antiphagocytosis (*cap*8E, *cap*8G), survival and virulence (*mgt*B), cytokine production and cytotoxicity (*ilp*A), infection and immune evasion (*icl*1), and fatty acid biosynthesis (*acp*P) (Table 2).

**TABLE 2:** Presence of virulence determinants in *Chryseobacterium indologenes* isolate.

Gene identifier	Putative gene	Encoding	Size (aa)	Reference species	aa identity (%)
NP_206868	*ure*G	Urease accessory protein UreG	212	*Helicobacter pylori*	74.24
NP_206872	*ure*B	Urease subunit β	573	*Helicobacter pylori*	66.26
NP_273273	*kat*A	Catalase KatA	495	*Neisseria meningitidis*	53.72
YP_094248	*kat*G	Catalase/peroxidase KatG	758	*Legionella pneumophila*	61.75
NP_644943	*cap*8E	Capsular polysaccharide synthesis enzyme Cap8E	344	*Straphylococcus aureus*	67.17
NP_644945	*cap*8G	Capsular polysaccharide synthesis enzyme Cap8G	379	*Straphylococcus aureus*	53.58
NP_464522	*clp*E	ATP-dependent protease	845	*Listeria monocytogenes*	50.39
NP_465991	*clp*P	ATP-dependent Clp protease proteolytic subunit	228	*Listeria monocytogenes*	52.38
NP_439034	*rff*G	dTDP-glucose 4,6-dehydratase	359	*Haemophilus influenzae*	51.03
NP_438515	*gal*E	UDP-glucose 4-epimerase GalE	339	*Haemophilus influenzae*	50.30
NP_933683	*ilp*A	MetQ/NlpA family lipoprotein adhesin IlpA	268	*Vibrio vulnificus*	55.56
YP_177728	*icl*1	Isocitrate lyase	426	*Mycobacterium tuberculosis*	61.15
NP_458740	*tvi*B	Vi polysaccharide biosynthesis UDP-N-acetylglucosamine C-6 dehydrogenase TviB	431	*Salmonella enterica*	53.72
NP_462662	*mgt*B	Magnesium-translocating P-type ATPase	890	*Salmonella enterica*	52.75
NP_540392	*acp*P	Acyl carrier protein	79	*Brucella melitensis*	58.67
YP_170388.1	*rfb*A	Glucose-1-phosphate thymidylyltransferase RfbA	287	*Francisella tularensis*	61.03
NP_878994	*wlb*B	O-antigen biosynthesis protein WlbB	210	*Bordetella pertussis*	53.33
YP_094724	*gro*L	Chaperonin GroEL	541	*Legionella pneumophila*	65.72
YP_169203.1	*tu*f	Elongation factor Tu	403	*Francisella tularensis*	67.25

### Genomic islands and other mobile genetic elements genes in *Chryseobacterium indologenes*

**FIGURE 7:**
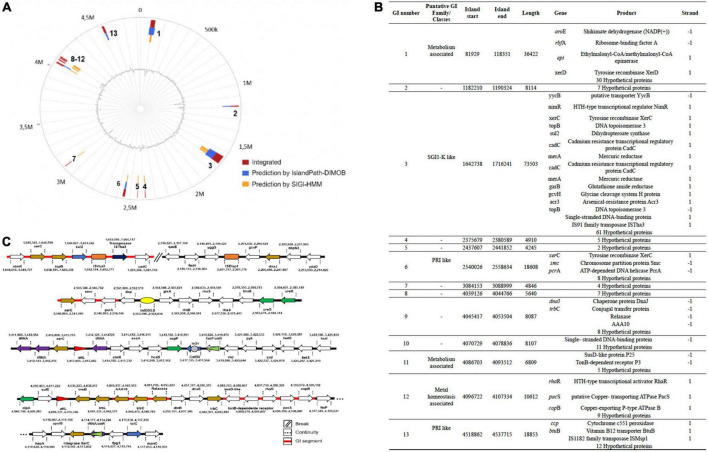
Schematic representation of the Genomic Islands (GIs) and Mobile Genetic Elements (MGEs) in *C. indologenes* strain. **(A)** Circular genomic representation of GIs. **(B)** Genetic composition of the GIs. **(C)** MGEs found in *C. indologenes*. Arrow: Light blue (resistance genes), dark blue (transposase genes), golden [elements associated with Integrative and Conjugative Element (ICE)], green (virulence genes), purple (tRNAs). Red/green triangles represent the attL and attR sequences, respectively. Orange squares represent Insertion Sequences. The yellow ellipse represents transposons. The gray circle represents integron.

Our analyses indicate that the *C. indologenes* genome contained 13 potential Genomic Islands (GI) (Figure 7A). These had some elements associated with one integrative and conjugative element (ICE), in the 8 and 12 GI regions, such as a copy of a *Trb*C, and a relaxase (*xer*C). The ICE, named ICE*Cind1*, contained further open reading frames (ORFs) encoding putative TrbL, TrbC (1 copy), TrwB, integrase, and it was bordered by a 16-bp direct repeat (attL; 5′-TTGTGGGTCCTGAGG-3′, and attR: 5′-TTGTGGGTCCTGAGGG-3′) at both ends. The attR was included in the 3’ end of the tRNA-Val(TAC). In GI, we also found a *sul2* gene encoding for resistance to sulfonamide, flanked by IS91-like element (ISVsa3 family transposase); an IS91 family transposase ISTha3; 2 copies of *xer*C; and other “accessory” sequences (Figures 7B,C).

Other elements linked to mobile genetic elements (MGEs) were present such as an insertion sequence (ISEIsp1), classified in the IS1595 family (ISPna2 group); and a transposon Tn5393. Sequence examination further indicated a region bordered by a 12-bp direct repeat (aatL 5′-ATTTTCTTAAAT-3 and attR 5′-ATTTTCTTAAAT-3) at both ends that contained the *cat*B3, a chloramphenicol acetyltransferase gene inserted in integron In31; one gene associated with fatty acid biosynthesis (*acp*P); a 3-phenylpropionate-dihydrodiol/cinnamic acid-dihydrodiol dehydrogenase (*hca*B) gene, a transcriptional regulator (*exs*A) gene; a quinone reductase (*chr*R) gene; and a tRNA (Figure 7B).

Through IslandViewer 4, we detected unaligned scaffolds that contained a ColRNAI type of replicons and genes associated with β-lactams (*bla*_*TEM–*116_, *bla*_*VEB–*15_, *bla*_*OXA–*209_), aminoglycosides (*aad*S), phenicol (*cat*B3), and macrolide (*erm*F) antibiotic resistance. CRISPR and phage elements were not detected in the *C. indologenes* genome.

## Discussion

Multidrug-resistant Gram-negative bacteria are usually associated with nosocomial infection and pose an important health problem for neonates admitted to neonatal intensive care units worldwide (9, 73). *Chryseobacterium indologenes* is an emerging nosocomial pathogen that has acquired clinical significance due to ubiquitous and intrinsical resistance to several antibiotics, life-threatening infection potential, and ability to persist in hospital settings (9, 74). The number of studies about *C. indologenes* is very limited, mainly in terms of their genomes. To build on current information, we performed a systematic genotypic characterization of a *C. indologenes* strain isolated from the cerebrospinal fluid of an infant.

Although there has been an increasing trend of *C. indologenes* bacteremia related to infants over the last few decades (5, 75, 76), most cases of infections caused by *C. indologenes* are still reported in adults (8, 10). The precise reason for fewer reports of *C. indologenes* infections in children remains unclear. Studies have suggested it may relate to the lower frequency of comorbidities in the pediatric population (77). Considering the pediatric population can present significant morbidity and immune dysfunction, we postulate that the relevance of *C. indologenes* isolated from clinical samples of infants can be challenging since novel molecular and phenotypic tests are providing rapid and accurate identification of many bacterial species, including glucose non-fermenting gram-negative bacilli (78, 79) such as *C. indologenes*.

*Chryseobacterium indologenes* often exhibit resistance to a wide variety of broad-spectrum antibiotics, including aminoglycosides, tetracyclines, chloramphenicol, macrolides, clindamycin, and teicoplanin; extended-spectrum penicillins; and first-, second- and third-generation cephalosporin, aztreonam, ticarcillin-clavulanate and the carbapenems (19, 23, 80). According to our phenotypic results, our *C. indologenes* strain was resistant to most of these antibiotics. It revealed a multidrug resistance profile, presenting *in vitro* resistance to a wide range of antibiotic classes including β-lactams, aminoglycosides, quinolone, glycylcycline, and polymyxin. Although some studies have reported that *C. indologenes* remains susceptible to trimethoprim/sulfamethoxazole, quinolones, minocycline, cefepime, ceftazidime, piperacillin-tazobactam, and rifampin (10, 18, 81, 82), our strain was resistant not only to piperacillin–tazobactam, ciprofloxacin, cefepime, and ceftazidime but also to colistin and tigecycline. Our findings are in accordance with other studies (3, 9, 24) that show an increasing trend in resistance of *C. indologenes* against most commonly used antimicrobial agents.

In recent years, whole-genome sequencing (WGS) has become an efficient method not only for understanding the evolution of a wide range of infectious pathogens, such as emerging bacteria, but also for outbreak surveillance and implementation of rapid infection control protocols (83). Thus, we decided to investigate the *C. indologenes* genome using WGS. Our draft genome revealed one circular chromosome that had a similar length (4,836 kb) to most of the sequenced genomes from *C. indologenes* deposited in NCBI. The genomic features of chromosomes annotated using RAST, eggNOG, and GO showed similar characteristics, presenting cellular processes that are essential to the bacteria (84). The genes related to the disease found in RAST and the defense mechanisms present in eggNOG analysis indicate our *C. indologenes* strain is associated with the multidrug resistance profile. Liang et al. (84) obtained similar results when they analyzed the genomic features and antimicrobial susceptibility patterns of the *Chryseobacterium arthrosphaerae* strain ED882-96 isolated from a patient in Taiwan.

To evaluate the taxonomic position and to confirm the species identification of our strain, we conducted a 16S rRNA analysis and found that our strain is affiliated with the species *C. indologenes* (GenBank: AM232813). Although 16S rRNA gene sequences are highly conserved among strains of the same bacterial species and are frequently used to identify and classify microorganisms, taxonomic classifications based only on the analysis of the 16S rRNA gene can lead to misclassifications in some cases (85). This happens because this analysis lacks sufficient discriminatory power to differentiate species in many genera, such as *Aeromonas*, *Bacillus, Pseudomonas*, *Streptococcus*, etc. (86, 87). Therefore, we used average nucleotide identity (ANI) and digital DNA–DNA hybridization (dDDH) to validate species identity, which confirmed that the most closely related species of our strain was *C. indologenes* (GCF_900460995.1).

The phylogenetic and corresponding taxonomic analysis is fundamental not only to establish the genetic novelty and the genotype–phenotype relationships of the isolates but also to identify the closest relatives of microorganisms within assembled genomes (88, 89). When we assessed the genetic relatedness between *C. indologenes* isolate and fifteen *C. indologenes* strains (90–92), we found that the five closest relatives of *C. indologenes* strains were isolated from clinical human samples. We then analyzed the distribution of shared gene families (sequence clusters) among the proteomes of our sample and four *C. indologenes* strains. Interestingly, our *C. indologenes* strain harbored the highest number of singleton genes compared to its closest four relatives. Some of the singleton genes were related to antibiotic resistance. The genomic variability and diversity among the isolates are highlighted by the analysis of singletons and non-core genes that may be acquired from distal lineages through horizontal gene transfer and represent the genetic source for the emergence of novel functions (89).

The biological functions of the KEGG pathway genes were analyzed to further characterize the genomic differences among five strains. KEGG pathway annotation revealed that most of the genes of the core genome are related to metabolism. Similar results were obtained in previous studies on *Chryseobacterium* genomes (93). Moreover, drug resistance genes that may contribute to the wide resistance of strain *C. indologenes* to antimicrobials were found in a unique genome. All these distinctive features of our *C. indologenes* isolate provide evidence of its genomic plasticity that may contribute to antibiotic resistance and environmental adaptation.

When investigating the genetic basis of multidrug resistance (MDR) profile in *C. indologenes*, we observed a high level of concordance between the phenotypic and the genotypic results. Our analysis showed that the resistance gene profile to β-lactams in *C. indologenes* isolate may be due to the carriage of *bla*_*CIA–*3_, *bla*_*IND–*13_, *bla*_*TEM–*116_, *bla*_*VEB*,_ and *bla*_*OXA–*209_. The *bla*_*CIA*_ and *bla*_*IND*_ genes have previously been identified in *C. indologenes* (94), and they usually provide intrinsic resistance to carbapenems and cephalosporins due to their production of class A β-lactamase (CIA) and class B carbapenem-hydrolyzing β-lactamase (IND) (23, 95, 96). The *_*bla*_*_*TEM–*116_ gene was described for the first time in Korea in a clinical isolate of *E. coli* (97). In Brazil, it was previously found in *Klebsiella pneumoniae* (98), *Vibrio parahaemo*lyticus (99), *Aeromonas hydrophila*, and *Aeromonas jandaei* (100). Studies have related that TEM-116 β-lactamase can confer resistance to ceftazidime, cefotaxime, and aztreonam (97, 101). The *bla*_*VEB–*15_ gene was first identified from genomic DNA of *E. coli* isolate that had reduced susceptibility to ceftazidime–avibactam (102). However, the *bla*_*VEB*_ group has been identified in a variety of species of *Enterobacteriaceae* and non-fermenting species such as *P. aeruginosa* and *Acinetobacter baumannii* (102). The VEB enzymes confer a high level of resistance to an expanded-spectrum cephalosporin (103, 104). The *bla*_*OXA–*209_ is a β-lactamase gene that was first described in *Riemerella anatipestifer* strain isolated from ducks and geese (105). It was also described more recently in pan-drug-resistant *Myroides odoratimimus* PR63039 strain isolated from a patient presenting with post-injury urinary tract infection (106). Although there were a few reports on the substrate profile of beta-lactamase class D OXA-209, we suggest this enzyme can contribute to the resistance of *C. indologenes* to β-lactam antibiotics.

Our analysis revealed the presence of genes that can mediate resistance to tigecycline (*tet(*X)), quinolones (*mcb*G and mutation in the *gyr*A and *gyr*B genes), polymyxin (*Hly*D, *Tol*C, and mutation in the lpxA gene), and aminoglycosides (*Ran*A and *Ran*B). Tet(X) is an enzyme capable of modifying the antibiotic tetracycline and its derivates, including tigecycline and glycylcycline (68, 107). Since the identification of the *tet*(X) gene from the obligately anaerobic *Bacteroides* spp. (108, 109), it has been reported among strains of *Enterobacteriaceae*, *Comamonadaceae, Flavobacteriaceae*, and *Moraxellaceae* (23, 110, 111). Resistance to quinolones can be conferred by mutations in quinolone-resistance-determining region (QRDRs) (gyrA, gyrB, parC, and parE subunits), efflux pumps (QepA and OqxAB), DNA topoisomerase protection protein Qnr, and quinolone acetyltransferase Aac (6′)-Ib-cr (23, 112, 113). Our strain displayed amino acid alternations at position 83 in GyrA (Ser83Tyr) (23, 114) and positions 425 and 473 in gyrB (Leu425 Ile and Lys473Arg) (23). These findings are in line with studies showing these mutations in *C. indologenes* (23). Furthermore, the *mcb*G gene, found in our *C. indologenes* strain, encodes a pentapeptide-repeat protein with 19.6% amino acid identity with QnrA (115). The mcbG protein protects DNA gyrase from the action of microcin B17 (MccB17) and some quinolones antibiotics (115, 116).

We also examined whether mutations in *pmr*C, *lpx*A, *lpx*C, and *lpx*D genes were associated with polymyxin resistance found in our strain. Our analysis revealed only a substitution in lpxA (Gly68Asp). Amino acid substitutions, frameshifts, or truncation of lpxD lpxA and lpxC have been demonstrated to lead to a complete loss of LPS (117). These changes may play a role in colistin resistance, as shown in clinical *A. baumannii* and *Chryseobacterium oranimense* isolates (117, 118).

Efflux systems have been described in several bacteria isolated from clinical specimens and can be related to multidrug resistance phenotypes (119). We identify genes associated with multidrug effux pumps, such as *Hly*D, *Tol*C, *Ran*A, and *Ran*B. HlyD belongs to the membrane fusion protein family (120) and forms a continuous channel by docking to the TolC. HlyD has been shown to contribute to polymyxin resistance in *A. baumannii* (121, 122). TolC, an outer-membrane channel protein, is often the final portal in the pathways of protein toxin transport or export of unwanted molecules, such as antibiotics (123–125). *Ran*A*Ran*B genes encode an efflux pump of the ABC efflux pump system. RanA alongside RanB mediates resistance to aminoglycosides in *Riemerella anatipestifer* (126), a member of the *Flavobacteriaceae* family.

Although some studies suggest that proteases and biofilm production may play an important role in the virulence of invasive infections caused by *C. indologenes* (8, 11), much remains unknown about virulence factors essential for pathogenicity and their mechanism during pathogenesis. Analyzing the virulence profiles of our strain, we found genes that encode conserved virulence factors, which have been previously identified in *C. indologenes* (127) and other pathogens (128). The virulence factors found were associated with oxidative stress such as catalase (*kat*A, *kat*G) (129, 130); adhesion to host cells and extracellular matrix components (elongation factor *Tu*) (131); colonization of a host organism and in maintenance of bacterial cells in tissues (*ure*B) (132); bacterial growth, stress tolerance, and biofilm formation (*clp*P) (133); modulating the expression of virulence determinants and metabolism-related factor (*clp*E) (134); cell envelope structure, swarmer cell elongation, and subsequent swarm motility (*rff*G) (135, 136); serum resistance and biofilm formation (*gal*E) (137–139); O-antigen nucleotide sugar biosynthesis (*wlb*B) (140); O-antigen-synthesis (*rfb*A) (141); polysaccharide biosynthesis (tviB) (142); capsular polysaccharide synthesis and antiphagocytosis (*cap*8E, *cap*8G) (143, 144); survival and virulence (*mgt*B) (145, 146), stress response (*gro*L) (147); induction of cytokine production, adhesion, and cytotoxicity (*ilp*A) (148, 149); and infection and immune evasion capacity (*icl*, *acp*) (150). Our data indicate that *C. indologenes* may be a highly virulent strain, presenting putative virulence factors related to structural functions, physiological activity, defense, or invasion that favor the course of pathogenesis.

Genomic Islands (GIs) are cluster genes in prokaryotic genomes of probable horizontal origin, which harbor components of mobile genetic elements (MGEs) that may be associated with mobilizing DNA (151, 152). GI regions often carry genes related to pathogenicity, symbiosis, metabolic, fitness, or resistance islands (153) that confer a selective advantage to the host bacterium (152). We searched the *C. indologenes* genome for the MGEs able to transfer genes between DNA molecules (insertion sequences, gene cassettes, integrons, and transposons) and for those able to transfer genes between cells (conjugative and mobilizable plasmids, and integrative and conjugative element (ICEs) (154). Our analysis showed that the most prevalent ORFs are hypothetical proteins, which are frequently found in GIs and ICEs (152). We also found a GI region with ICE features (ICE*Cind*1) that contained copies of a relaxase-encoding gene; genes related to a type IV secretion system such as three conjugative transfer protein-encoding genes (1 copy *trb*L and 2 copies of *trb*C) (155, 156); a gene related to essential integral membrane protein (*Trw*B), important for the conjugation process (157); and a gene encoding a site-specific integrase (*xer*C), which ensures the site-specific chromosomal integration of the ICE as well as effective excision of the element, where it may be aided by an excisionase or recombination directionality factor (158–160). In addition, we found a transposon Tn5393 that usually carries the *str*A and *str*B genes, responsible for the resistance to streptomycin (161, 162). However, these resistance genes were not present in this region. The *sul2* gene, encoding for resistance to sulfonamide, was flanked by ISVsa3 family transposase (IS91-like element). This has been previously described in a plasmid (pEPMS-18199) from *Edwardsiella piscicida* (163). The *cat* variant genes usually participate in the composition of gene cassette or integron, and confer the ability of antibiotic resistance (164). *Chryseobacterium indologenes* contained a *cat*B3, a chloramphenicol acetyltransferase gene, inserted in integron In31. Interestingly, Laraki et al. (165) showed that *cat*B6, a chloramphenicol acetyltransferase−encoding allele of the catB family, may be inserted in this integron (In31). The authors also observed it decreased the *in vitro* antibiotic susceptibilities of *Pseudomonas aeruginosa* strains.

Although WGS allows the analysis of large datasets using *in silico* plasmid typing methods, short reads from popular high-throughput sequencers can be difficult to assemble. Therefore, complete plasmid sequences may not be accurately reconstructed (166). When antimicrobial resistance genes are localized on incomplete contigs, it is uncertain whether they are localized on plasmid or chromosome (167). In our *C. indologenes* strain, most resistance genes (*bla*_*TEM–*116_, *bla*_*VEB–*15_, *bla*_*OXA–*209_, *aad*S, *cat*B3, *erm*F) detected in singletons and the replicon (ColRNAI_DQ298019) were located on unaligned scaffolds. These findings partially corroborate the results of Evans et al. (168), who found a ColRNAI plasmid harboring genes associated with β-lactams (*bla*_*OXA–*9_, *bla*_*TEM–*1*A*_), chloramphenicol (*cat*A1) antibiotic resistance. Furthermore, studies have shown that *cat*B3 is one member of the gene cassette *aac*A7-*cat*B3-*aad*B-*oxa*2-*orf*D. It can be mobilized by the integron-encoded DNA integrase and plays a role in chloramphenicol resistance of plasmid pBWH301 (169, 170). However, this complete gene cassette was not found in our analysis.

The scarcity of data on the properties of clinical isolates of *C. indologenes* makes it challenging to characterize the transmission and evolution of this pathogen. Therefore, we believe that our detailed data of the WGS will contribute to the understanding of the genomic diversity, pathogenic potential, and multidrug resistance profile presented by *C. indologenes*. In addition, our data may guide future public health policy and MDR *C. indologenes* infection control.

## Data availability statement

The datasets presented in this study can be found in online repositories. The names of the repository/repositories and accession number(s) can be found in the link: https://www.ncbi.nlm.nih.gov/bioproject/?term=PRJNA830910. Bioproject accession id is PRJNA830910.

## Ethics statement

The studies involving human participants were reviewed and approved by the Committee of Ethics in Human Research of the Federal University of São Carlos (no. 1.595.268). In this work, *C. indologenes* and the anonymous archival data related patient age, gender, and sample type were obtained from the Central Laboratory of Public Health of Tocantins (LACEN/TO, data’s owner). Patient consent was not required since the data presented in this study do not relate to any specific person or persons. Written informed consent from the participants or their legal guardian/next of kin was not required to participate in this study in accordance with the national legislation and the institutional requirements. Permission to conduct the present study was obtained from the Health Department of the State of Tocantins (Secretaria da Saúde do Estado do Tocantins – SESAU) and LACEN/TO.

## Author contributions

MSFD, RLF, EBC, GGS, LCC, PML, and ASC performed the experiments. CCMF, LTC, and AFC aided with the bioinformatics analysis. MCSP, MSFD, EBC, and AP-S wrote the manuscript and analyzed the data. MCSP conceived and supervised the study. All authors contributed to the article and approved the submitted version.

## Footnotes

1
https://www.bioinformatics.babraham.ac.uk/projects/fastqc/

2
https://www.bioinformatics.babraham.ac.uk/projects/trim_galore/

3
https://cge.cbs.dtu.dk/services/PlasmidFinder/

4
https://rast.nmpdr.org/

5
http://eggnog-mapper.embl.de/

6
https://www.genome.jp/kegg/

7
https://www.ezbiocloud.net/tools/orthoani

8
https://ggdc.dsmz.de/ggdc.php#

9
https://discover.nci.nih.gov/cimminer/

10
https://orthovenn2.bioinfotoolkits.net/

11
https://cge.cbs.dtu.dk/services/ResFinder/

12
https://card.mcmaster.ca/

13
http://blast.ncbi.nlm.nih.gov

14
https://www.pathogenomics.sfu.ca/islandviewer/upload/

15
https://www-is.biotoul.fr/

16
https://tncentral.ncc.unesp.br/

17
https://galaxy.pasteur.fr/

18
https://crisprcas.i2bc.paris-saclay.fr/CrisprCasFinder/Index

19
https://phaster.ca/

20
https://bioinfo-mml.sjtu.edu.cn/ICEfinder/ICEfinder.html

21
https://www.ncbi.nlm.nih.gov/sra
